# Quantification of cyanogenic glycoside content and correlational pest pressure in American elderberry genotypes

**DOI:** 10.3389/fpls.2026.1959734

**Published:** 2026-10-26

**Authors:** Ronald J. Myers, Sydney D. Mendenhall, Elizabeth Prenger, Andrew L. Thomas, Lloyd W. Sumner

**Affiliations:** 1 Division of Biochemistry, Interdisciplinary Plant Group, Christopher S. Bond Life Sciences Center, College of Agriculture, Food and Natural Resources, University of Missouri, Columbia, MO, United States; 2 Division of Plant Science and Technology, Southwest Research, Extension, and Education Center, University of Missouri, Mount Vernon, MO, United States; 3 Division of Plant Science and Technology, University of Missouri, Columbia, MO, United States; 4 The Center for Agroforestry, Anheuser-Busch Natural Resources Building, University of Missouri, Columbia, MO, United States

**Keywords:** American Elderberry, cyanogenic glycosides, herbivory, mass spectrometry, Sambucus

## Abstract

Plants have evolved an array of defensive molecules to survive damaging environmental factors. Some plants utilize cyanogenic glycosides that can be rapidly hydrolyzed into highly toxic hydrogen cyanide upon mechanical injury, inducing an unfavorable feeding environment for generalist herbivores in the process termed plant cyanogenesis. Little is known regarding plant cyanogenesis in the rapidly emerging specialty crop American elderberry (*Sambucus nigra* subsp. *canadensis*). With American elderberry being susceptible to a variety of pests and pathogens, understanding whether cyanogenic glycosides within this species correlate with herbivory and pathogen resistance is crucial for cultivar development. We utilized ultra-high-pressure liquid chromatography coupled to a triple quadrupole tandem mass spectrometer (UHPLC-MS/MS) to evaluate quantitative and qualitative diversity in cyanogenic glycoside content in leaf tissues across a collection of twelve American elderberry genotypes. Total and individual cyanogenic glycoside concentrations were then compared to field performance data to better understand a potential basis for pest and pathogen incidence. Our study revealed that pest pressure is variable among genotypes and that correlations exist between increasing concentrations of both total and individual cyanogenic glycosides within leaves to reduced herbivory and disease damage incidence in field-grown plants. Therefore, leaf cyanogenic glycoside concentration constitutes a potential marker for developing herbivory- and pathogen- resistant American elderberry plants for cultivation. We further report phytochemical evidence that American elderberry produces both aliphatic and aromatic cyanogenic glycosides, which is extremely rare. Additional investigation into the emerging genome sequence of American elderberry provided reinforcing genetic evidence for the presence of both biosynthetic pathways.

## 1 Introduction

Plants are sessile organisms that have evolved a wide array of chemically defensive molecules and responses to survive in the presence of damaging biotic and abiotic environmental factors, including insects and disease (Chen, 2008; Vega-Muñoz et al., 2020; Vlot et al., 2020). Cyanogenic glycosides (CNGs) are one common class of defensive molecules that are synthesized as specialized plant natural products/secondary metabolites within over 3,000 plant species and have established roles in protection against herbivory and pathogens (Gleadow and Møller, 2014; Piršelová and Jakubčinová, 2025; Groover et al., 2026). Cellular disruption via mechanical injury, such as damage incurred as a result of chewing insects or pathogens, results in the breakdown of subcellular compartmentalization within plant tissues, allowing CNGs stored within the plant vacuole to be released into the intracellular and extracellular spaces (Morant et al., 2008). Cyanogenic glycosides then undergo hydrolysis via intracellular plant β-glucosidase enzymes followed by rapid conversion of the nitrile group either spontaneously or through S-hydroxynitrile lyases in a process that releases hydrogen cyanide (HCN) (Morant et al., 2008; Yulvianti and Zidorn, 2021). This two-step process has been termed plant cyanogenesis, and it is reported to provide anti-herbivory effects against generalist predators through consumption of the toxic HCN moiety (Poulton, 1990; Boter and Diaz, 2023; Katamreddy et al., 2025). CNGs have also been implicated in having beneficial roles within resistance to multiple abiotic environmental stressors, including drought and frost events, and within plant nitrogen regulation as nitrogen storage molecules/reservoirs (Piršelová and Jakubčinová, 2025). Negative effects of CNGs on the plant itself are generally well tolerated, as CNGs are typically sequestered away from native plant β-glucosidases until severe damage occurs (Piršelová and Jakubčinová, 2025). The primary negative effect of CNG biosynthesis is similar to other secondary metabolites in that it induces an additional carbon cost on the plant, potentially reducing carbon availability for sugars and having an effect on growth (Herms and Mattson, 1992). Roles for CNGs in herbivory and microbial resistance have been well established within the literature for crop plants, with reductions in generalist herbivory being seen in sorghum, cassava, and lima bean as a result of higher cyanogenic potential (Jones, 1962; Bellotti and Riis, 1994; Tattersall et al., 2001; Riis et al, 2003; Ballhorn et al., 2009; Krothapalli et al., 2013; Pinto-Zevallos et al., 2016; Piršelová and Jakubčinová, 2025). There is not a conclusive threshold at which plants are known to reach a high enough cyanogenic potential to reduce herbivory across different species. However, the generally reported range is approximately 200 to 700 ppm in fresh tissue within cassava and lima bean, respectively (Riis et al., 2003; Ballhorn et al., 2009).

American elderberry (*Sambucus nigra* subsp. *canadensis*) is a crop species that is known to produce cyanogenic glycosides (Buhrmester et al., 2000; Appenteng et al., 2021; Kasote et al., 2024). This species is a rapidly emerging specialty crop natively found and commercially grown within midwestern and eastern North America (Huchteman et al., 2025). American elderberry belongs to the same genus as the more widely studied European elderberry (*Sambucus nigra* subsp. *nigra*), and similarly to European elderberry, it is highly sought for use within juices, wines, syrups, and dietary supplements because of its high concentration of antioxidant- and health-promoting compounds, such as terpenoids, flavonoids, and anthocyanins (Senica et al., 2016; Thomas et al., 2020; Dwikarina et al., 2024). Previous reports highlight differences in anthocyanin content between American and European elderberries (Lee and Finn, 2007). The elderberry market is poised to continue growing by as much as 8.8% on a yearly basis worldwide for the foreseeable future, with American elderberry expected to be a significant portion of this growth (Cai et al., 2024). Thus, the evaluation and identification of American elderberry genotypes that are tolerant to environmental stressors, such as herbivory and pathogen attack, are of interest to crop breeders as these would allow for the development of genotypes that are less susceptible to yield losses in this emerging industry. Our knowledge from other crop species suggests that higher CNG levels could potentially reduce herbivory pressure, making the naturally occurring American elderberry leaf CNG concentrations a potential marker for genotype development (Jones, 1962; Bellotti and Riis, 1994; Tattersall et al., 2001; Riis et al, 2003; Ballhorn et al., 2009; Krothapalli et al., 2013; Piršelová and Jakubčinová, 2025).

Unlike other crop species, there is much that remains unknown regarding cyanogenesis in American elderberry. Previous studies have identified that American elderberry produces up to five chemically distinct cyanogenic glycosides within its vegetative tissues (Appenteng et al., 2021; Kasote et al., 2024). Additionally, randomly chosen roadside populations have been shown to be variable regarding their cyanogenic potential, but it is not clear whether these populations were genetically different (Buhrmester et al., 2000). Outside of these three reports, there are no studies that have investigated cyanogenesis in American elderberry. None of the available literature has investigated CNG content in this species across a range of American elderberry genotypes. In the only genotype comparative analysis that has been previously performed, Appenteng et al. compared the cyanogenic potential of the two genotypes ‘Ozone’ and ‘Ozark’ and found that total cyanogenic potential appeared to be similar across both genotypes (Appenteng et al., 2021). Cyanogenic content in leaf tissue was not compared in their study. Subsequently, no studies have attempted to determine whether is an association between cyanogenic glycoside content in this species and incurred herbivory pressure. Thus, there is a knowledge gap regarding whether different genotypes of American elderberry produce different concentrations of cyanogenic glycosides, and if so, whether different levels of cyanogenic glycosides are associated with altered herbivory pressures in field grown plants.

Several species of arthropod pests are known to damage and defoliate American elderberry, including eriophyid mite (*Phyllocoptes* spp.), Japanese beetle (*Popillia japonica*), sawfly (*Tenthrado grandis*), and elder borer beetle (*Desmocerus palliatus*), among others (Thomas et al., 2015; Warmund and Amrine, 2015; Moore, 2024). American elderberry is also susceptible to fungal diseases, such as elderberry rust (*Puccinia* spp.), along with both fungal and bacterial leafspot diseases (Hensel, 2017; Warmund et al., 2019). Previous studies have investigated pest incidence among varieties of American elderberry genotypes and found that susceptibility to Japanese beetle and eriophyid mite damage varies among genotypes (Finn et al., 2008; Thomas et al., 2015). It is not clear if there is a chemical basis for the differences in herbivory susceptibility amongst these genotypes. Large-scale studies on the presence or absence of cyanogenic glycosides in American elderberry have suggested that a small number of American elderberry populations are highly cyanogenic, but that most populations appear to have low cyanogenic potential or be acyanogenic (Buhrmester et al., 2000). It is possible that differences in cyanogenic potential across genotypes influence susceptibility to Japanese beetle and eriophyid mite in this species.

The role of cyanogenic glycosides in American elderberry is not well described, but recent research has indicated that in addition to being cyanogenic, this species may contain an unusual combination of chemically distinct CNGs (Appenteng et al., 2021). The production of both aromatic and aliphatic cyanogenic glycosides does not typically occur within the same plant, as they require different biochemical pathways with unique Cytochrome P450 enzymes (Busk and Møller, 2002; Jørgensen et al., 2011; Boter and Diaz, 2023). The lack of multiple, chemically distinct cyanogenic glycosides occurring simultaneously in most cyanogenic plants has resulted in a knowledge gap regarding whether a significant difference between the effectiveness of different CNGs on reducing herbivory in plants can occur. Thus, it is currently unknown if each individual CNG within a plant’s cyanogenic profile is uniquely suited to reducing pest pressure to a specific pest species. However, it was recently reported that American elderberry contains both aromatic (i.e., dhurrin, amygdalin, (R)-prunasin, (S)-prunasin) and aliphatic (i.e., linamarin) CNGs (Appenteng et al., 2021; Kasote et al., 2024). It is currently unknown whether American elderberry contains genes encoding both aliphatic and/or aromatic cyanogenic glycoside pathways, but previous phytochemical studies suggest that both chemical pathways may be present (Appenteng et al., 2021). The presence of these chemically divergent CNGs may be unique to American elderberry, as dhurrin and linamarin have not been identified in the closely related blue elderberry (*Sambucus nigra* subsp. *cerulea*) or European elderberry subspecies (Uhl and Mitchell, 2024). This specific combination of CNGs is highly unusual, as dhurrin is most commonly found in the Poaceae family and linamarin is typically found only in Asteraceae, Euphorbiaceae, Linaceae, and Fabaceae plant families (Vetter, 2000; Uhl and Mitchell, 2024). A genetic basis for both aromatic and aliphatic CNG biosynthesis in American elderberry is a crucial gap that has previously not been possible to address. However, efforts to sequence this species are ongoing and an unpublished American elderberry genome has been generated at the University of Missouri. This resource has made it possible to begin utilizing bioinformatics analyses to investigate whether this species is truly unique in its cyanogenic glycoside profile. To our knowledge, the effectiveness of chemically distinct CNGs on reducing the incidence of various types of pests and pathogens has never been explored, as most cyanogenic plant species produce only one or two types of CNGs and studies have generally focused on total cyanogenic potential rather than absolute quantification of each individual CNG (Uhl and Mitchell, 2024; Piršelová and Jakubčinová, 2025). By studying both the total and individual cyanogenic glycoside concentrations within genotypes of this species, American elderberry provides a potentially unique opportunity for discovering differential effectiveness of chemically distinct cyanogenic metabolites on herbivory pressures.

Our study aimed to quantify individual and total cyanogenic glycoside content across multiple American elderberry genotypes and correlate cyanogenic content with acquired field herbivory data to better understand a potential metabolic basis for differences in pest incidence in this emerging crop species. Specifically, we asked the following questions to gain a better understanding of cyanogenesis in American elderberry: (1) Are the five cyanogenic glycosides recently reported in Appenteng et al. present across a wide variety of American elderberry genotypes, and if so, what are their concentrations in leaf tissue? (2) Can American elderberry genotypes that are native to different regions of the midwestern United States be differentiated into low and high cyanogenic potential varieties based on total cyanogenic glycoside concentrations within their leaf tissue? (3) Does the American elderberry genome contain genes encoding for both aromatic and aliphatic cyanogenic glycoside pathways? (4) Does increased total and/or individual cyanogenic glycoside content within American elderberry genotypes negatively correlate with herbivory (i.e., Japanese beetle and eriophyid mite) and/or pathogen (i.e., leaf spot) damage pressures measured in the field?

## 2 Materials and methods

### 2.1 Chemicals and reagents

Commercially available cyanogenic glycoside standards were purchased for use in cyanogenic glycoside quantification. Amygdalin, linamarin, dhurrin, prunasin, and umbelliferone were purchased from Sigma-Aldrich (St. Louis, Missouri, USA). Sambunigrin was acquired from Santa Cruz Biotechnology (Dallas, Texas, USA). Optima™ LC/MS grade methanol, acetonitrile, and formic acid were purchased from Fisher Chemical (Hampton, New Hampshire, USA) for use during mass spectral analyses. Milli-Q water used during chemical extractions and instrumental analyses was obtained from an in-house Milli-Q water purification system (MilliporeSigma, Burlington, MA, USA).

### 2.2 American elderberry genotypes and planting conditions

Twelve American elderberry genotypes originating from eight different states in the midwestern and eastern regions of the United States were established and grown at two University of Missouri research facilities in 2022: (1) the Southwest Research, Extension, and Education Center (SWC) at Mount Vernon, MO, and (2) the Horticulture and Agroforestry Research Farm (HARF) in New Franklin, MO. The plantings were part of a larger genotype x environment study performed across five different locations throughout the midwestern region of the United States. The SWC research site was located at the geographic coordinates 37.074677, -93.879393 in USDA Hardiness Zone 7a, with Creldon silt loam soil, an average low temperature of -5 to 0°F, and 47.75 inches of yearly rainfall (Moore, 2024). HARF was located at the coordinates 39.019269, -92.760997 in a 6b hardiness zone with both Sibley silt loam and Menfro silt loam soils, an average low temperature of -5 to 0°F, and 41.98 inches of yearly rainfall (Moore, 2024). Soil tests were performed prior to the initial planting in 2022 and at the beginning of the growing season in 2023 and 2024 (Moore, 2024; **Supplementary Table S1**). The soil tests measured multiple parameters, including pH, phosphorus (P), potassium (K), calcium (Ca), magnesium (Mg), neutralizable acidity (NA), sulfur (SO_4_-S), zinc (Zn), organic matter (OM), manganese (Mn), iron (Fe), copper (Cu), and cation exchange capacity (CEC). Each genotype that was selected for the study was chosen based on its high level of performance within its native region. Additionally, each genotype was selected based on being native to disparate regions from one another, as there is extremely limited genotypic information available in American elderberry. Thus, cultivars have not yet been proven to be different genetically but are phenotypically different and expected to have differing genetics with their geographic separation (Moore, 2024). The twelve genotypes investigated during the study, along with their U.S. state of origin, are as follows: Anoka (Minnesota), Dandy (Wisconsin), Delight (Wisconsin), East Grove (Iowa), Ranch (Kansas), Bob Gordon (Missouri), Coop (Missouri), Rogersville (Missouri), Pocahontas (Arkansas), Barber (Oklahoma), Cherokee (Oklahoma), and Hamilton (Florida). Hardwood cuttings of each genotype were rooted in the SWC greenhouse in March of 2022, propagated by dipping the proximal end in TakeRoot^®^ Rooting Hormone (indole-3-butyric acid), and grown in pots containing Pro-Mix potting mix for 4–6 weeks before transplantation in the field. The plants at HARF and SWC were clonally propagated, ensuring that the genetics of each genotype would be identical on a per-site basis for subsequent analyses. Plantings were performed in six replications in a completely randomized design in May 2022. Permeable weed barrier fabric was used, with irrigation supplied as needed during establishment. Plants received light dormant pruning in early 2023 and 2024 and were cut back to the ground in the winter of 2025. Plot setups and full descriptions of the field preparation and management strategies have been previously described (Moore, 2024).

### 2.3 Collection of data for pest and disease incidence in American elderberry

Pest and disease incidence data were collected at the SWC in June and July of the 2023 and 2024 growing seasons, with leaf damage incurred as a result of Japanese beetle, eriophyid mite, and leaf spot being documented on a 1–5 scale. Measurements were collected manually by the same individual during the 2023 and 2024 growing seasons at SWC. Each whole number is representative of a 25% damage incidence range (1 = no damage, 2 = < 25% damage, 3 = 25-50% damage, 4 = 51-75% damage, and 5 = > 75% damage). Differentiation between fungal and bacterial leaf spot diseases can be challenging in American elderberry, as it is commonly infected by both and each type has similar symptoms (Hensel, 2017). Thus, we considered leaf spot damage as a combined measure of any leaf spot damage that was present on the plant leaves. Similar methodology with a slightly different severity scale design (4-point scale) was employed to measure eriophyid mite damage during the 2025 growing season at HARF (0 = no damage, 1 = mild damage, limited to leaves, 2 = moderate damage, leaves and some fruit may be affected, 3 = severe damage affecting both the leaves and fruit). Measurements at HARF were taken by a different individual than those performed at SWC. The HARF location was utilized only during the 2025 growing season to achieve a better understanding of a potential relationship between genotypic cyanogenic glycoside content and reduced eriophyid mite damage.

### 2.4 American elderberry sample acquisition and preparation

During the growing season of 2023, whole leaf samples from four different plants of each genotype within the SWC planting were collected and flash frozen. Additional whole leaf samples were taken from the SWC during 2024, but these were ground and heat dried following their collection, making them less suitable for CNG quantification (Sheikh et al., 2021). Thus, we utilized freshly frozen tissue solely from the 2023 SWC site for our analyses to achieve a more accurate representation of CNG concentrations on a per genotype basis. Samples were initially stored at -20 °C, then soon after maintained at -80 °C until laboratory analyses. Leaves that were utilized for analyses were of similar size and developmental stage. In all cases, the third leaflet pair from the branch tip was chosen for extraction. Immediately preceding chemical extraction, samples were divided into identical leaflet pairs of similar age and size on dry ice and lyophilized using a Labconco FreeZone freeze dryer (Kansas City, MO, USA). The initial shelf temperature of the freeze dryer was set to hold for 3 hours at -40 °C. The shelf temperature was then programmed to move to a second segment of -15 °C for 72 hours during primary drying and third segment of 10 °C for 21 hours during secondary drying. Lyophilization was performed to minimize degradation and simplify the extraction process, as previous reports have indicated that the concentrations of cyanogenic glycosides in fresh tissue versus lyophilized tissue are comparable (Appenteng et al., 2021). Following lyophilization, samples were stored in Falcon tubes at room temperature until further processing.

### 2.5 Cyanogenic glycoside extraction

Extraction of cyanogenic glycoside content from leaf tissue was performed similarly and as previously described to our recently developed in-house protocol, with slight modification (Kasote et al., 2024). Lyophilized leaf samples (10 ± 0.05 mg) were weighed into 4-dram glass vials (Thermo Fisher Scientific, Waltham, Massachusetts, USA) for chemical extraction. 1.0 mL of 80% methanol/20% water containing an internal standard (umbelliferone, 18 µg/mL) was added directly to the sample to begin the initial extraction. Each sample was then vortexed for 30 seconds and sonicated for 10 minutes prior to being agitated at room temperature for 18 hours at approximately 70 rpm on an orbital shaker. Following agitation, samples were centrifuged for 20 minutes at 3500 rpm and the supernatant (800 µL) was transferred into a new Eppendorf tube, with care taken to avoid disruption of the cellular debris pellet. The samples were then centrifuged a second time at 13,000 rpm for 15 minutes. After the second centrifugation step, the supernatant was transferred to a clear 2 mL glass SureSTART™ autosampler vial (Thermo Fisher Scientific) and the extracts were stored at -20 °C until mass spectral analyses.

### 2.6 UHPLC-MS/MS method parameters, analyses, and quantification

Analytical conditions for simultaneous quantification of five individual cyanogenic glycosides (amygdalin, dhurrin, linamarin, sambunigrin, and prunasin) using UHPLC-MS/MS were nearly identical to those described in Kasote et al. (2024). In short, a Waters ACQUITY Premier UPLC system (Milford, MA, USA) coupled to a Waters Xevo TQ Absolute triple quadrupole mass spectrometer (Milford, MA, USA) was utilized for chromatographic separations and mass spectral analyses. An ACQUITY UPLC HSS T3, 1.8 µm, 2.1 x 150 mm column (Milford, MA, USA) was employed for chromatographic separations at a column temperature of 45 °C. Gradient elution was used in the separation of the cyanogenic glycosides, consisting of Mobile Phase A (2 mM ammonium formate in water), Mobile Phase B (100% acetonitrile), and the following elution profile: initial equilibration at 1% B; 0–12 min, 1–12% B; 12–12.5 min, 12–95% B; 12.5–14.5 min, 95% B; 14.5–15 min, 95–5% B; 15–18 min, 5–1% B. A total of 2 µL was injected from each sample during each mass spectral analysis, and each analysis was 18 minutes in total. The mass spectrometer was operated in positive electrospray ionization (+ESI) mode, with cone voltage, collision energy, and quantifier and qualifier mass-to-charge ratio transitions for multiple reaction monitoring (MRM) chosen based on previously described values (Kasote et al., 2024). To enable quantification of unknown cyanogenic glycoside concentrations within samples, an external standard curve consisting of the following concentrations of each cyanogenic glycoside and an internal standard (18 µg/mL umbelliferone) was generated at the beginning of each instrumental analysis series: 1 ng/µL, 100 ng/µL, 500 ng/µL, 1 µg/µL, 5 µg/µL, 10 µg/µL, and 20 µg/µL, 50 µg/µL, and 100 µg/µL. Waters MassLynx software (version 4.2, Milford, MA, USA) and embedded TargetLynx software (version 4.2, Milford, MA, USA) were used for data processing and compound quantification. Quantification was done similarly as performed in Kasote et al., 2024. Standard curves were created on a daily basis at the beginning of each sample batch during the instrumental analyses to account for any drift in the instrumental response. Standard curve values were obtained from the series of standards by integrating the area under each peak for every monitored transition ion pair within the standard series. Normalization was performed following acquisition of peak areas by dividing the peak area of each analyte standard by the peak area of the internal umbelliferone standard to generate a response ratio. Obtained values were plotted as the standard concentration versus the response ratio and a linear regression equation was generated. The quantifier transitions outlined in Kasote et al., 2024 for each individual cyanogenic glycoside were utilized to create a standard curve for each analyte of interest. R-squared values have been included within the supplemental data (**Supplementary Table S2**). Quantification of the unknown CNG levels within each sample was performed by acquiring the response ratio for each quantifier peak in the MRM chromatogram and solving for the unknown value using the linear regression equation of the corresponding standard curve for each analyte. All quantification was performed via interpolation, with the response ratios falling within the linear portion of the standard curve. The three points of the standard curve that were most closely adjacent to the response ratio for the unknown sample were used to generate a more precise linear regression equation for quantification. The final CNG concentration in µg per gram of leaf tissue was determined by taking the total amount of the CNG within the analyzed sample divided by the amount (in grams) of tissue used in the analysis. Total CNG content was quantified by adding together the determined concentrations for each of the five individual CNGs in each individual leaf sample for the 12 tested American elderberry genotypes.

### 2.7 tBLASTn homology analyses

Homologous genes for the production of both aliphatic or aromatic cyanogenic glycosides in American elderberry were putatively identified using tBLASTn bioinformatics analyses. An unpublished American elderberry reference genome sequenced from the “Bob Gordon” genotype was obtained from the University of Missouri as a .fna file containing 4,436 segmented sequences and 11,470,569,852 total letters. A local BLAST nucleotide database was created from the .fna file using the most recent version of BLAST+ (ncbi-blast-2.17.0+). Amino acid sequences for known Cytochrome P450s involved in cyanogenic glycoside biosynthesis in *Sorghum bicolor* (Sorghum), *Manihot esculenta* (Cassava), and *Prunus mume* (Plum) were collected from NCBI Gene and saved in FASTA formats for subsequent analyses. Specifically, amino acid sequences from Cytochrome P450 enzymes with the GenBank protein accession numbers of BAP15888.1, AAC39318.1, Q6XQ14.1, NP_001410932.1, AAF27289.1, and AAF27290.1 were utilized for the determination of potential homologous genes in American elderberry. Each protein sequence was compared against each possible translation of the American elderberry nucleotide sequence using tBLASTn within BLAST+ (Altschul et al., 1997). The top hit for each analysis was reported, with a quantitative score (in bits), expectation value, percent identity, percent similarity, and gap percentage being included.

### 2.8 Statistical analyses

One way ANalysis Of VAriance (ANOVA) followed by Tukey’s *post-hoc* tests were performed for statistical comparisons of both the cyanogenic glycoside content within the evaluated genotypes and for determination of differences in herbivory or pathogen damage incurred among genotypes during the 2023, 2024, and 2025 field studies. Letters correspond to a statistically significant difference of at least P < 0.05. The results for each cyanogenic glycoside quantification experiment are displayed as box-and-whisker plots, where the borders of the plots represent the 25^th^ and 75^th^ percentiles of the obtained data. All collected data are included and represented as individual points within each plot, with the horizontal line representing the median and “X” indicating the mean value. Results consist of data collected from four biological replicates of each genotype, with two independent observations/measurements being taken for each analysis from individual leaf samples of the same plant (8 total measurements, 4 plants utilized). Cyanogenic glycoside concentration values that fell below the limit of quantification (LOQ) were replaced using mean imputation with the mean of observed values for the specific variable of interest for statistical purposes. We used this approach as a complete data set was needed to perform statistical analyses and simple mean imputation is a commonly used method for eliminating missing values due to LOQ detection limits in targeted metabolomics studies (Wei et al., 2018). Data values within the text are reported as the mean value ± standard deviation. Correlation coefficients were obtained by performing Pearson correlation tests using R within RStudio (version 2025.09.1 + 401). Negative Pearson correlation coefficients are representative of an inverse relationship between two continuous variables.

## 3 Results

### 3.1 Relative quantitative abundance of individual cyanogenic glycosides varied in 12 American elderberry genotypes

Amygdalin was present in each of the genotypes that we tested (**Figure 1A**). The Coop genotype had much higher amygdalin content relative to the others, having a mean value of 94.01 ± 18.76 µg/g of this CNG within the leaf tissue (**Figure 1A**; **Supplementary Table S2**). The remaining genotypes were found to have concentrations that clustered closely together, with the lowest amygdalin abundance genotype, Ranch, having 0.56 ± 0.04 µg/g and the second highest abundant genotype, Hamilton, having 0.62 ± 0.06 µg/g of this CNG (**Figure 1A**; **Supplementary Table S2**). Coop was the only genotype that had a significantly higher amygdalin concentration than each of the other genotypes (**Figure 1A**; **Supplementary Table S2**).

**Figure 1:**
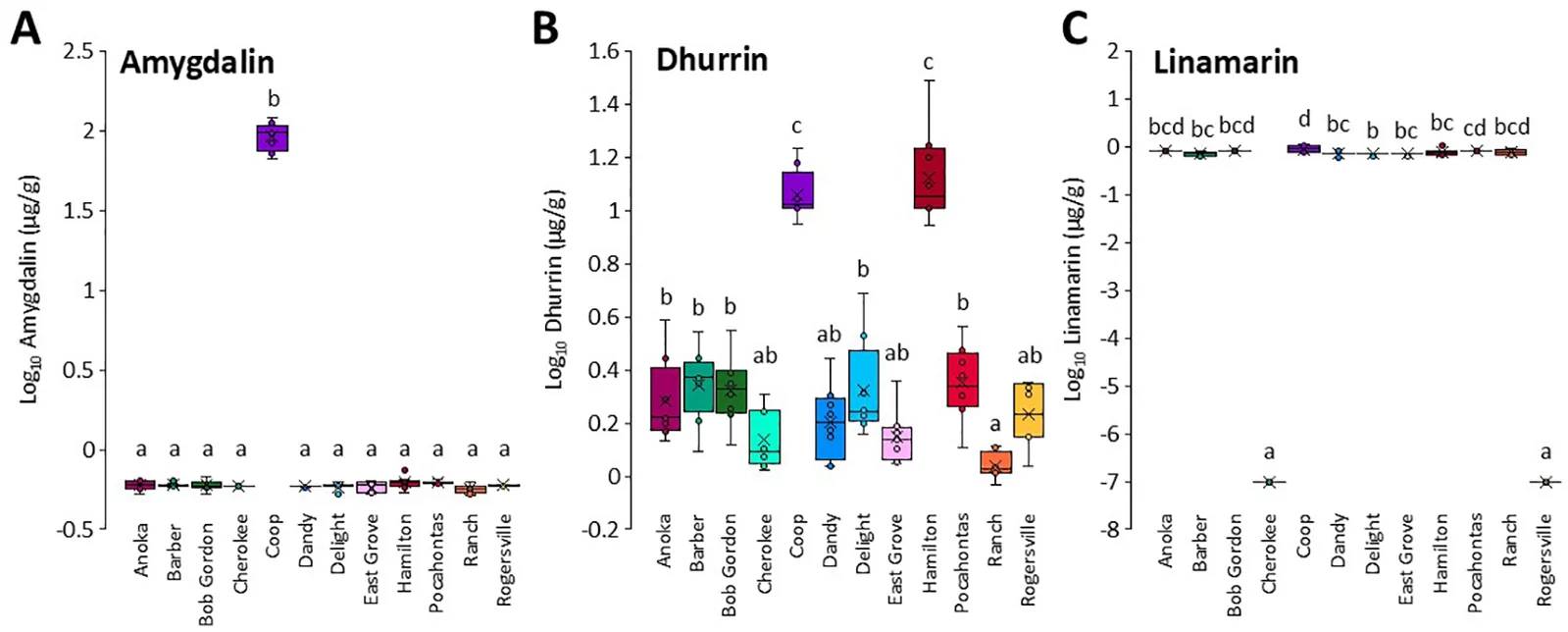
Individual cyanogenic glycoside content for three of the identified cyanogenic glycosides within twelve individual genotypes of *Sambucus nigra* subsp. *canadensis* (American elderberry). **(A)** Box plots showing the base-10 logarithmic quantified levels of amygdalin in each of the tested American elderberry genotypes. **(B)** Same as A, except for dhurrin. **(C)** Same as A and B, except for linamarin. Statistical significance was determined by one-way ANOVA (different letters above bars indicate significance at P < 0.05; N = 4, with two independent observations for each analysis). Borders of each box and whisker plot correspond to the 25^th^ and 75^th^ percentiles of the data. Each collected data point is indicated within each plot. The horizontal line is representative of the median of the data while the “X” is representative of the mean. Whiskers are indicative of 1.5 times the minimum and maximum of the mean. Abbreviations: µg/g, microgram per gram; Log_10_, logarithmic base 10.

Dhurrin was also found to be present within the leaves of each of the twelve genotypes that we tested (**Figures 1B, C**). We examined its relative abundance to amygdalin within American elderberry leaves and found that leaf dhurrin concentrations are higher relative to amygdalin concentrations across the majority of the genotypes that were tested (**Figure 1B**; **Supplementary Table S2**). Coop and Hamilton had significantly higher dhurrin concentrations than each of the other genotypes (**Figure 1B**; **Supplementary Table S2**). Hamilton contained the highest levels of dhurrin (14.51 ± 7.26 µg/g), with Coop following closely behind (11.72 ± 2.84 µg/g) (**Figure 1B**; **Supplementary Table S2**). Ranch and Cherokee contained the lowest levels of this CNG at 1.09 ± 0.12 µg/g and 1.42 ± 0.38 µg/g, respectively. Coop was the only genotype to have a lower dhurrin concentration relative to amygdalin concentration within the leaf tissue.

Linamarin was also found to be present, though there were no genotypes that contained dramatically higher concentrations of linamarin relative to the others (**Figure 1C**; **Supplementary Table S2**). Linamarin content was slightly higher in abundance relative to amygdalin across most of the tested genotypes; however, the levels of linamarin were more comparable to those of amygdalin than to dhurrin (**Figure 1C**; **Supplementary Table S2**). Linamarin levels were lowest in Barber at 0.72 ± 0.07 µg/g and highest in Coop at 0.91 ± 0.13 µg/g (**Figure 1C**; **Supplementary Table S2**). No linamarin was detected in the Cherokee and Rogersville genotypes.

### 3.2 Differentiation between the stereoisomers prunasin and sambunigrin

Prunasin (R-prunasin) was found to be present in 11/12 of our genotypes of interest while sambunigrin (S-prunasin) was found within all genotypes (**Figures 2A, B**; **Supplementary Table S2**). We found that Coop had a very high concentration of sambunigrin within its leaves (18,816.73 ± 2515.24 µg/g) relative to the other genotypes (0.67 – 1.71 µg/g; **Figure 2A**; **Supplementary Table S2**). Coop was an extreme outlier, but there were also statistically significant differences between sambunigrin concentrations for the remaining genotypes (**Figure 2A**; **Supplementary Table S2**). East Grove was found to contain the most sambunigrin (1.71 ± 1.10 µg/g) relative to the other genotypes (with the exception of Coop) while Barber had the least (0.67 ± 0.12 µg/g) (**Figure 2A**; **Supplementary Table S2**). Additionally, we analyzed the concentration of prunasin within the twelve genotypes and found that Hamilton had the highest levels of this cyanogenic glycoside (27.92 ± 32.44 µg/g) while Coop had the lowest levels (not detectable). Prunasin had the highest number of individually statistically significant groups among different genotypes compared to the other CNGs that were tested (**Figure 2B**; **Supplementary Table S2**).

**Figure 2:**
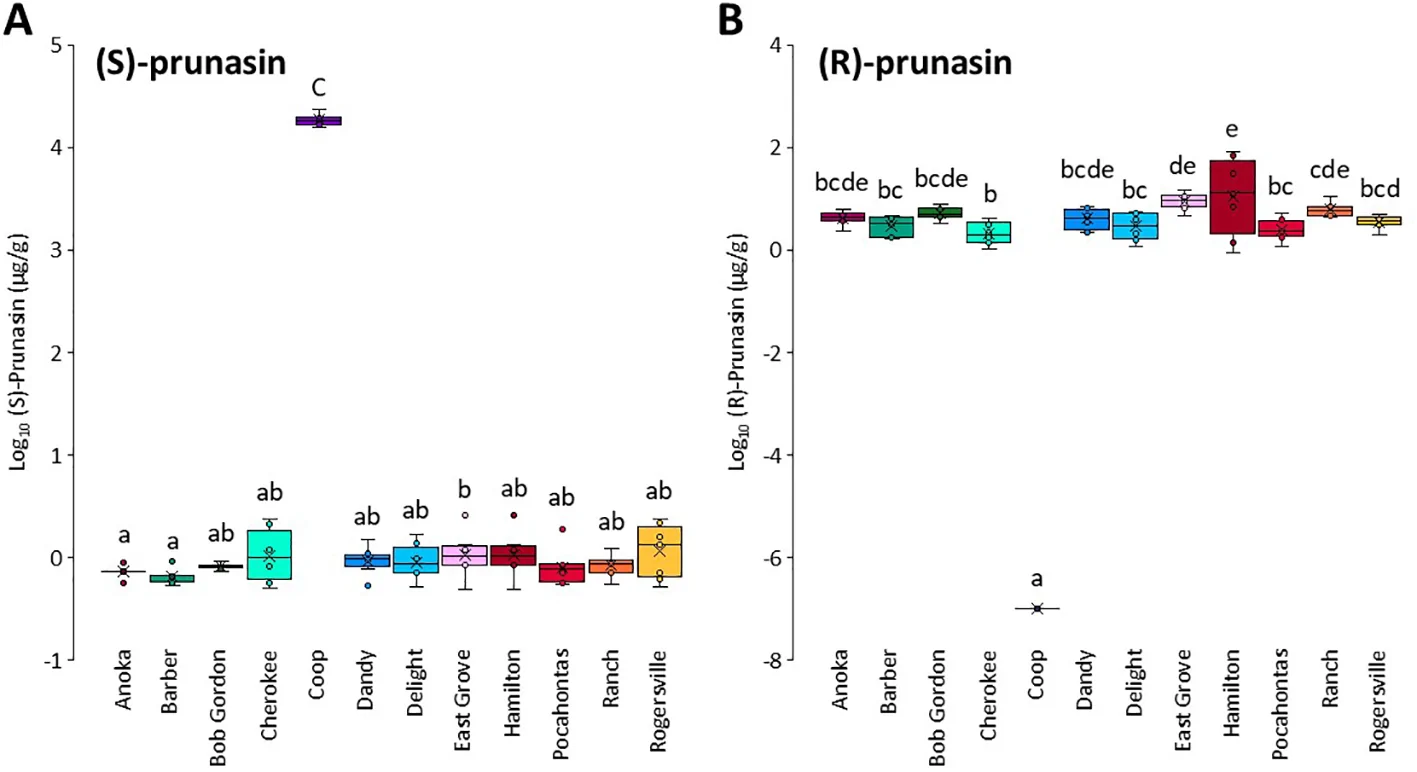
Individual cyanogenic glycoside content for the stereoisomeric metabolites (S)-prunasin and (R)-prunasin within twelve individual genotypes of *Sambucus nigra* subsp. *canadensis* (American elderberry). **(A)** Box plots showing the base-10 logarithmic quantified levels of (S)-prunasin in each of the tested American elderberry genotypes. **(B)** Same as A, except for (R)-prunasin. Statistical significance was determined by one-way ANOVA (different letters above bars indicate significance at P < 0.05; N = 4, with two independent observations for each analysis). Borders of each box and whisker plot correspond to the 25^th^ and 75^th^ percentiles of the data. Each collected data point is indicated within each plot. The horizontal line is representative of the median of the data while the “X” is representative of the mean. Whiskers are indicative of 1.5 times the minimum and maximum of the mean. Abbreviations: µg/g, microgram per gram; Log_10_, logarithmic base 10.

### 3.3 Total cyanogenic glycoside content is genotype specific

Coop was found to have the greatest concentration of total cyanogenic glycoside content within the leaf by a large margin (18,923.38 ± 2529.21 µg of CNGs per gram of lyophilized tissue; **Figure 3**; **Supplementary Table S2**). This concentration was over 400-fold higher than the second highest genotype, Hamilton, which had a total quantifiable CNG content of 44.37 ± 37.78 µg/g (**Figure 3**; **Supplementary Table S2**). Following Hamilton, the levels of CNG content dropped considerably, with East Grove containing the third-highest levels of CNGs (13.13 ± 3.51 µg/g) (**Figure 3**; **Supplementary Table S2**). The Coop, Hamilton, and East Grove genotypes had significantly higher concentrations of CNGs than the remaining genotypes (**Figure 3**; **Supplementary Table S2**). The genotypes with the lowest total levels of CNGs were Rogersville and Cherokee, with 6.78 ± 1.22 and 4.95 ± 1.70 µg/g of CNGs, respectively (**Figure 3**; **Supplementary Table S2**).

**Figure 3:**
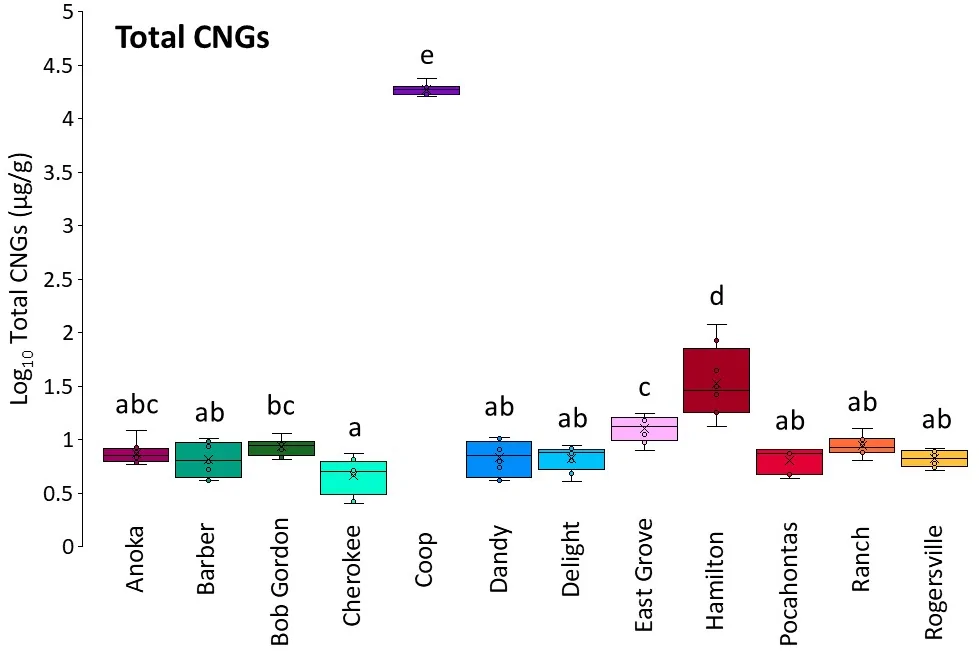
Total combined cyanogenic glycoside content for five individually identified cyanogenic glycoside metabolites within twelve individual genotypes of *Sambucus nigra* subsp. *canadensis* (American elderberry). Box plots showing the base-10 logarithmic quantified levels of cyanogenic glycosides in each of the tested American elderberry genotypes. Statistical significance was determined by one-way ANOVA (different letters above bars indicate significance at P < 0.05; N = 4, with two independent observations for each analysis). Borders of each box and whisker plot correspond to the 25^th^ and 75^th^ percentiles of the data. Each collected data point is indicated within each plot. The horizontal line is representative of the median of the data while the “X” is representative of the mean. Whiskers are indicative of 1.5 times the minimum and maximum of the mean. Abbreviations: CNGs, cyanogenic glycosides; µg/g, microgram per gram; Log_10_, logarithmic base 10.

### 3.4 American elderberry contains putatively identified homologous cytochrome P450 gene sequences for both aliphatic and aromatic cyanogenic glycoside biosynthesis

Each of the six different cytochrome P450 enzyme amino acid sequences that we investigated via tBLASTn comparisons appeared to have at least partially homologous counterparts within the American elderberry reference genome (**Table 1**). The expectation values were all found to be 4e^-61^ or less, indicating high statistical significance that corresponds to very strong evidence of potential homology (**Table 1**). Bit scores for each top hit were found to be within ranges considered to be a strong predictor of homology, being at 204 or higher for each query (**Table 1**). Identical sequence identities ranged from 42 – 62%, while positive sequence similarity scored as high as 79% (**Table 1**). Minimal gaps were missing from the potentially homologous regions, as five of the six tested amino acid sequences had a 1% or less gap in the reference genome (**Table 1**). Taken together, the data from the outputs of the tBLASTn analyses suggest that homologous genes for both aromatic (dhurrin, prunasin) and aliphatic (linamarin) cyanogenic glycoside production are likely present within American elderberry. The strongest hits for these sequence homologies were mostly found on either chromosome 3, 15, or 16 of the American elderberry genome (**Supplementary Table S3**).

**Table 1:** Putatively identified homologous enzymes for aromatic and aliphatic cyanogenic glycoside synthesis in American elderberry.

Enzyme	Length (AA)	Organism	CNG Type	Chemical Pathway	Score (Bits)	E Value	Identity	Similarity	Gaps
Cytochrome P450 71AN24	526	*Prunus mume*	Aromatic	Prunasin	228	5E-119	120/205 (59%)	151/205 (74%)	3/205 (1%)
Cytochrome P450 CYP71E7	511	*Manihot esculenta*	Aliphatic	Linamarin	204	1E-87	94/206 (46%)	141/206 (68%)	2/206 (1%)
Cytochrome P450 CYP79A1	558	*Sorghum bicolor*	Aromatic	Dhurrin	289	2E-82	129/208 (62%)	165/208 (79%)	0/208 (0%)
Cytochrome P450 CYP79D1	542	*Manihot esculenta*	Aliphatic	Linamarin	279	2E-79	129/209 (62%)	164/209 (78%)	2/209 (1%)
Cytochrome P450 CYP79D2	541	*Manihot esculenta*	Aliphatic	Linamarin	274	1E-77	125/209 (60%)	160/209 (77%)	2/209 (1%)
Cytochrome P450 CYP71E1	531	*Sorghum bicolor*	Aromatic	Dhurrin	225	4E-61	116/274 (42%)	166/274 (61%)	8/274 (3%)

Tabulated data for tBLASTn results of known enzymes involved within cyanogenic glycoside production in other cyanogenic plant species versus the American elderberry reference genome. CNG, cyanogenic glycoside; AA, amino acid; E value, expect value.

### 3.5 Resistance to disease and pest incidence is variable across different American elderberry genotypes

During the 2023 growing season, we found that the Coop, Bob Gordon, and Hamilton genotypes had the least leaf damage while the Ranch genotype suffered the most damage from Japanese beetles (**Table 2A**; **Supplementary Table S4**). Eriophyid mite damage was less prominent, with minimal damage and no statistically significant differences between genotypes being seen (**Table 2A**; **Supplementary Table S4**). Leaf spot was observed to cause moderate levels of damage to most of the genotypes in the study (**Table 2A**; **Supplementary Table S4**). Hamilton and Pocahontas appeared to have the lowest incidence of leaf spot damage, while East Grove had the most (**Table 2A**; **Supplementary Table S4**). Hamilton uniquely experienced low levels of both herbivory damage (Japanese beetle, and mite) and leaf spot damage during the 2023 growing season.

**Table 2:** Pest and disease damage incidence in twelve individual genotypes of Sambucus nigra subsp. canadensis (American elderberry).

A. 2023 SWC							B. 2024 SWC							C. 2025 HARF		
Genotype	Japanese Beetle		Eriophyid Mite		Leaf Spot Disease		Genotype	Japanese Beetle		Eriophyid Mite		Leaf Spot Disease		Genotype	Eriophyid Mite	
	Est	SE	Est	SE	Est	SE		Est	SE	Est	SE	Est	SE		Est	SE
Anoka	1.25 abcd	0.17	1.00 a	0.00	2.08 ab	0.08	Anoka	1.17 ab	0.11	1.08 a	0.08	2.67 bc	0.14	Anoka	0.40 abc	0.22
Barber	2.08 de	0.18	1.00 a	0.00	2.33 abc	0.14	Barber	1.67 c	0.14	1.00 a	0.00	2.33 ab	0.14	Barber	1.00 bcd	0.00
Bob Gordon	1.08 ab	0.08	1.00 a	0.00	2.08 ab	0.08	Bob Gordon	1.00 a	0.00	1.17 ab	0.00	2.42 ab	0.14	Bob Gordon	1.00 bcd	0.00
Cherokee	2.08 de	0.18	1.17 a	0.11	2.42 abc	0.14	Cherokee	1.25 abc	0.13	1.83 c	0.20	2.75 bc	0.17	Cherokee	2.00 e	0.28
Coop	1.00 a	0.00	1.00 a	0.00	2.75 bc	0.24	Coop	1.08 ab	0.08	1.33 abc	0.14	2.17 ab	0.11	Coop	0.40 ab	0.19
Dandy	2.25 e	0.17	1.00 a	0.00	2.67 bc	0.18	Dandy	1.08 ab	0.08	1.67 bc	0.18	2.67 bc	0.14	Dandy	0.67 abc	0.19
Delight	1.75 abcde	0.17	1.00 a	0.00	2.25 abc	0.13	Delight	1.08 ab	0.08	1.08a	0.08	3.17 c	0.20	Delight	1.00 cde	0.00
East Grove	1.25 abcd	0.13	1.00 a	0.00	2.92 c	0.18	East Grove	1.00 a	0.00	1.00 a	0.00	2.75 bc	0.13	East Grove	1.17 bcde	0.15
Hamilton	1.17 abc	0.11	1.00 a	0.00	1.67 a	0.14	Hamilton	1.00 a	0.00	1.00 a	0.00	2.08 ab	0.08	Hamilton	0.00 a	0.00
Pocahontas	2.00 cde	0.17	1.08 a	0.08	1.57 abc	0.25	Pocahontas	1.50 bc	0.14	1.00 a	0.00	2.25 ab	0.13	Pocahontas	0.00 a	0.00
Ranch	2.67 e	0.21	1.00 a	0.00	2.42 abc	0.14	Ranch	1.75 c	0.13	1.08 ab	0.08	2.25 ab	0.13	Ranch	1.67 de	0.19
Rogersville	1.33 bcde	0.14	1.00 a	0.00	2.17 abc	0.16	Rogersville	1.17 abc	0.11	1.33 ab	0.18	1.83 a	0.20	Rogersville	1.17 bcde	0.15

(A) Tabulated data of American elderberry pest and disease damage occurrence during the 2023 growing season from the Southwest Research, Extension, and Education Center (SWC; Mount Vernon, Missouri). (B) Same as A, except for the 2024 growing season. (C) Tabulated data of incurred eriophyid mite damage in American elderberry genotypes at the Horticulture and Agroforestry Research Farm (HARF; New Franklin, Missouri) during the 2025 growing season. Statistical significance was determined by one-way ANOVA (different letters above bars indicate significance at P < 0.05; N ≥ 5). Damage was scored on a 1 (no damage) – 5 (severe damage) scale during the 2023 and 2024 growing seasons and a 0 (no damage) – 3 (severe damage) scale during the 2025 growing season. Abbreviations: Est, Estimate; SE, Standard error; SWC, Southwest Research, Extension, and Education Center; HARF, Horticulture and Agroforestry Research Farm.

During 2024 at SWC, there was a similar pattern seen among the genotypes regarding Japanese beetle damage, as Coop, Bob Gordon, and Hamilton once again had minimal Japanese beetle damage during the season (**Table 2B**; **Supplementary Table S4**). However, there was less pest pressure from Japanese beetles overall, making it more difficult to see differences between genotypes (**Table 2B**; **Supplementary Table S4**). Ranch again suffered the most damage to Japanese beetles during the season (**Table 2B**; **Supplementary Table S4**). Although pressure from mites was still relatively low during 2024, there were observable differences in damage between genotypes that were not clear during 2023 (**Tables 2A**, **1B**; **Supplementary Table S4**). Cherokee and Dandy incurred the most mite damage during this season, while Hamilton, East Grove, Pocahontas, and Barber incurred the least (**Table 2B**; **Supplementary Table S4**). Leaf spot damage and genotype susceptibility during 2024 appeared similar to 2023, with Hamilton and Pocahontas having relatively low levels of damage, but with Rogersville showing the least amount of damage for this year (**Table 2B**; **Supplementary Table S4**).

To determine with higher confidence whether there were any differences between mite damage among genotypes, we measured leaf damage incidence to eriophyid mite for each genotype during the 2025 growing season on a similar 0–3 scale at HARF. The low levels of mite incidence at SWC had made it difficult to differentiate between genotypes that may experience less damage to mites. Larger differences in mite pressure were seen at HARF, providing an opportunity to clarify these differences (**Supplementary Table S5**). It is not entirely clear whether these differences were due to varying perceptions of the measurement takers, the slightly different scale that was utilized at HARF relative to SWC, or a true increase in mite pressure relative to SWC. The increased range of damage seen during 2025 at HARF allowed for better differentiation between putatively resistant and non-resistant genotypes (**Table 2C**). The additional eriophyid mite data collected at HARF in 2025 showed that Hamilton and Pocahontas displayed the least amount of mite damage while Cherokee and Ranch experienced the highest levels of damage (**Table 2C**; **Supplementary Table S5**). This was similar to the 2024 data collected for mites at SWC, but the ability to distinguish between greater degrees of damage incidence made the comparison much clearer (**Tables 2B**, **C**; **Supplementary Table S5**). Additionally, Anoka, Coop, and Dandy appeared to sustain less damage to mites at this location when compared to the other genotypes (**Table 2C**).

### 3.6 Correlation of field performance data with cyanogenic glycoside content in American elderberry genotypes

Correlation analyses performed on data collected for the 2023 and 2024 growing seasons at SWC displayed a potential relationship between increasing CNG levels and reduced pest damage in American elderberry, albeit with low correlation coefficient values for each relationship. For the 2023 season, a highly significant but weak Pearson correlation coefficient of -0.33 was found between total CNGs and Japanese beetle damage (**Table 3A**). This correlation was not as strong or significant during the 2024 season; however, reduced herbivory/lower Japanese beetle pressure during this season made observable differences between genotypes more challenging (**Table 3B**; **Supplementary Table S4**). No relationship between reduced eriophyid mite damage and total CNG content was seen for 2023 or 2024, but the overall mite pressure during these seasons was extremely low (**Tables 2A, B**, **3A, B**; **Supplementary Table S4**). When mite damage was more abundant during the 2025 season at HARF, a weakly negative but nonsignificant relationship was found between total CNG content and mite damage. During the 2024 growing season at SWC, there was a very slight but significant negative correlation between total CNGs and leaf spot damage, but this was not consistent across seasons (**Tables 3A, B**).

**Table 3:** Pearson correlations between levels of cyanogenic glycosides and arthropod and pathogen damage incurred within leaves of tested *Sambucus nigra* subsp. *canadensis* (American elderberry) genotypes.

A. 2023 SWC				B. 2024 SWC				C. 2023 SWC			
CNG	Condition	Pearson	P-Value	CNG	Condition	Pearson	P-Value	CNG	Condition	Pearson	P-Value
Total CNG	Japanese beetle Mites Leaf spot	-0.33 -0.07 0.15	6.72e^-5^ 0.3724 0.0751	Total CNG	Japanese beetle Mites Leaf spot	-0.15 0.01 -0.17	0.0769 0.8594 0.0414	Total CNG	Mites	-0.21	0.0779
Amygdalin	Japanese beetle Mites Leaf spot	-0.27 -0.04 -0.16	0.0009 0.6101 0.0209	Amygdalin	Japanese beetle Mites Leaf spot	-0.11 0.07 -0.14	0.2049 0.2195 0.0897	Amygdalin	Mites	-0.21	0.0785
Dhurrin	Japanese beetle Mites Leaf spot	-0.40 -0.08 -0.16	8.26e^-7^ 0.3701 0.0614	Dhurrin	Japanese beetle Mites Leaf spot	-0.22 -0.10 -0.22	0.0093 0.2195 0.0080	Dhurrin	Mites	-0.48	2.58e^-5^
Linamarin	Japanese beetle Mites Leaf spot	-0.03 -0.20 0.03	0.7020 0.0190 0.6829	Linamarin	Japanese beetle Mites Leaf spot	0.02 -0.35 0.11	0.7920 2.00e^-5^ 0.1949	Linamarin	Mites	-0.44	0.0001
(S)-Prunasin	Japanese beetle Mites Leaf spot	-0.28 -0.04 0.21	0.0006 0.6143 0.0121	(S)-Prunasin	Japanese beetle Mites Leaf spot	-0.13 0.08 -0.14	0.1221 0.3194 0.1034	(S)-Prunasin	Mites	-0.21	0.0790
(R)-Prunasin	Japanese beetle Mites Leaf spot	0.24 0.02 -0.20	0.0041 0.7745 0.0145	(R)-Prunasin	Japanese beetle Mites Leaf spot	0.08 -0.10 0.12	0.3181 0.2399 0.1392	(R)-Prunasin	Mites	-0.28	0.0203

(A) Pearson correlations between levels of cyanogenic glycosides and Japanese beetle, eriophyid mite, or fungal leaf spot damage measured across twelve tested American elderberry genotypes during the 2023 growing season at the Southwest Research, Extension, and Education Center (SWC; Mount Vernon, MO). (B) Same as A, except for data collected for Japanese beetle, eriophyid mite, and fungal leaf spot damage during the 2024 growing season. (C) Same as A and B, except for eriophyid mite damage at the Horticulture and Agroforestry Research Farm (HARF) during the 2025 growing season (New Franklin, MO). Abbreviations: CNGs, cyanogenic glycosides; SWC, Southwest Research, Extension, and Education Center; HARF, Horticulture and Agroforestry Research Farm. Pearson correlation values ranging between ± 0.20 - 0.39 were considered to be weak correlations while values ranging from ± 0.40 -0.69 were considered to be moderate correlations.

When accounting for each CNG individually during the 2023 season, there were both weak and moderate correlations between the concentrations of individual CNGs and herbivory damage that were experienced by the tested genotypes (**Table 3A**). A weakly significant relationship was found between amygdalin (correlation value of -0.27), and sambunigrin (correlation value of -0.28) concentrations and reduced Japanese beetle damage in 2023 (**Table 3A**). Additionally, a relationship between dhurrin and reduced Japanese beetle damage was also found during this season, being moderate and significant with a Pearson correlation value of -0.40 (**Table 3A**). Unlike the other CNGs, prunasin had a positive correlation between its concentration and Japanese beetle damage (**Table 3A**). Linamarin was the only CNG that did not have a relationship with Japanese beetle damage during 2023; rather, linamarin was weakly but significantly associated with reduced mite damage during this season (**Table 3A**). Relationships between individual CNGs and leaf spot were inconsistent, as sambunigrin and amygdalin had a weakly positive and significant correlation but prunasin had a significant and weak negative relationship with leaf spot damage (**Table 3A**).

Fewer significant relationships between individual CNGs and herbivory damage were found during the 2024 growing season. No significant relationships were seen between amygdalin, sambunigrin, nor prunasin and herbivory or leaf spot damage (**Table 3B**). Similarly to 2023, there was a negative relationship between linamarin and mite damage, though this effect was more pronounced (moderate) in 2024 (**Table 3B**). Additionally, the relationships between dhurrin and Japanese beetle and leaf spot followed a similar pattern to 2023, with weakly negative and significant relationships between Japanese beetle or leaf spot damage and dhurrin being found during 2024 (**Table 3B**).

The mite damage data collected for the 2025 season at HARF displayed more significant relationships between individual CNG content and mites than seen during the previous seasons (**Table 3C**). Linamarin and dhurrin were found to have a moderate relationship with reduced eriophyid mite damage, having significant Pearson correlation coefficients of -0.44 and -0.49, respectively (**Table 3C**). Prunasin was also found to be weakly and significantly correlated with reduced mite damage (**Table 3C**). Though amygdalin and sambunigrin both displayed weakly negative relationships with mite damage during this season, the relationships were not significant (**Table 3C**).

Using our collected data, we synthesized a hierarchy of both the total and individual CNG content within each genotype grouped from highest to lowest CNG concentration (**Table 4**). Most genotypes that were high in total overall CNG content ranked highly across each individually measured CNG (**Table 4**). However, there were exceptions, with Pocahontas scoring low on total CNG concentrations but being on the upper end of the genotype rankings for amygdalin, dhurrin, and linamarin content (**Table 4**). Additionally, we compiled a summarization table of genotypes, their origin, total cyanogenic glycosides levels, and damage measurements experienced during each growing season to facilitate comparison between genotypes (**Table 5**).

**Table 4:** A ranking of total and individual cyanogenic glycoside levels for each tested American elderberry genotype.

Total CNG		Amygdalin		Dhurrin		Linamarin		(S)-Prunasin		(R)-Prunasin	
1	Coop	1	Coop	1	Coop	1	Coop	1	Coop	1	Hamilton
2	Hamilton	2	Anoka		Hamilton	2	Pocahontas	2	East Grove	2	East Grove
3	East Grove		Barber	2	Anoka		Anoka	3	Bob Gordon	3	Ranch
4	Bob Gordon		Bob Gordon		Barber	3	Bob Gordon		Cherokee	4	Bob Gordon
5	Anoka Barber		Cherokee		Bob Gordon		Ranch		Dandy		Anoka
			Dandy		Delight	4	Barber		Delight		Dandy
6	Dandy Delight Pocahontas Ranch Rogersville		Delight		Pocahontas		Dandy		Hamilton	5	Rogersville
			East Grove	3	Cherokee		East Grove		Pocahontas	6	Barber
			Hamilton		Dandy		Hamilton		Ranch		Delight
			Pocahontas		East Grove		Delight		Rogersville		Pocahontas
			Ranch		Rogersville	5	Cherokee	4	Anoka	7	Cherokee
7	Cherokee		Rogersville		Ranch	6	Rogersville		Barber	8	Coop

Statistical significance was determined by one-way ANOVA (different numbers within each ranking indicate groups with statistical significance at P < 0.05; N = 4, with two independent observations for each analysis). Abbreviations: CNG, cyanogenic glycosides.

**Table 5:** A summary of genotypes, genotype origins, total cyanogenic glycoside content, and damage experienced on a per-season basis in American elderberry.

Genotype	Origin	Total CNG (µg/g)	Japanese beetle damage	Eriophyid mite damage	Leaf spot disease damage
Anoka	Minnesota	7.64	2023: 1.25 2024: 1.17	2023: 1.00 2024: 1.08 *2025: 0.40	2023: 2.08 2024: 2.67
Barber	Oklahoma	6.89	2023: 2.08 2024: 1.67	2023: 1.00 2024: 1.00 *2025: 1.00	2023: 2.33 2024: 2.33
Bob Gordon	Missouri	8.75	2023: 1.08 2024: 1.00	2023: 1.00 2024: 1.17 *2025: 1.00	2023: 2.08 2024: 2.42
Cherokee	Oklahoma	4.95	2023: 2.08 2024: 1.25	2023: 1.17 2024: 1.83 *2025: 2.00	2023: 2.42 2024: 2.75
Coop	Missouri	18,923.38	2023: 1.00 2024: 1.08	2023: 1.00 2024: 1.33 *2025: 0.40	2023: 2.75 2024: 2.17
Dandy	Wisconsin	7.18	2023: 2.25 2024: 1.08	2023: 1.00 2024: 1.67 *2025: 0.67	2023: 2.67 2024: 2.67
Delight	Wisconsin	6.30	2023: 1.75 2024: 1.08	2023: 1.00 2024: 1.08 *2025: 1.00	2023: 2.25 2024: 3.17
East Grove	Iowa	13. 13	2023: 1.25 2024: 1.00	2023: 1.00 2024: 1.00 *2025: 1.17	2023: 2.92 2024: 2.75
Hamilton	Florida	44.37	2023: 1.17 2024: 1.00	2023: 1.00 2024: 1.00 *2025: 0.00	2023: 1.67 2024: 2.08
Pocahontas	Arkansas	6.65	2023: 2.00 2024: 1.50	2023: 1.08 2024: 1.00 *2025: 0.00	2023: 1.57 2024: 2.25
Ranch	Kansas	8.95	2023: 2.67 2024: 1.75	2023: 1.00 2024: 1.08 *2025: 1.67	2023: 2.42 2024: 2.25
Rogersville	Missouri	6.78	2023: 1.33 2024: 1.17	2023: 1.00 2024: 1.33 *2025: 1.17	2023: 2.17 2024: 1.83

The * denotes damage index values collected at HARF. Abbreviations: CNG, cyanogenic glycosides; µg/g, microgram per gram.

## 4 Discussion

American elderberry has been reported to produce multiple and chemically diverse cyanogenic glycosides (Appenteng et al., 2021), and we were able to identify five chemically distinct CNGs within American elderberry (Kasote et al., 2024). We measured the CNGs content in 12 genotypes, and our findings suggest that CNG concentrations within American elderberry leaf tissues are highly dependent on the genotype and are produced in either similar or greater concentrations than previously described values in other American elderberry tissues, including fruits, skins, and stems (Appenteng et al., 2021). Additionally, certain genotypes may have extremely high concentrations of CNGs (i.e, sambunigrin/S-prunasin; **Figure 2A**) relative to other CNGs within the genotype and/or other genotypes. Herbivory and pathogen attack are prominent biotic stressors, making the selection of genotypes with endogenous insect resistance highly desirable. Understanding associations of leaf CNG content is important for potentially improving herbivory resistance in American elderberry, but no previous studies have investigated the CNG composition across a larger set of genotypes. Additionally, no studies have analyzed the role of structurally diverse CNGs on their correlation with protection against different agricultural pests, as plants that are capable of producing multiple types of CNGs are extremely rare.

American elderberry appears to be unique in that it contains a broader diversity of cyanogenic glycosides than many other plant species. Aromatic and aliphatic cyanogenic glycosides occurring in the same plant are exceedingly rare, as they are biosynthesized from separate amino acid pathways that require unique Cytochrome P450 enzymes (Busk and Møller, 2002; Jørgensen et al., 2011; Boter and Diaz, 2023). Early work on cyanogenic glycosides within American elderberry had detected sambunigrin (S-prunasin), but more recent work has suggested that there are also low levels of other cyanogenic glycosides in this species, including both aromatic and aliphatic CNGs (Buhrmester et al., 2000; Appenteng et al., 2021). This discrepancy may be due to differences in the sensitivities of the measurement methodologies used, as modern mass spectrometry technologies are much more sensitive than those available in 2000. Our work was able to identify and quantify each of the previously identified cyanogenic glycosides discovered in Appenteng et al., 2021. Additionally, using an evolving reference genome for American elderberry, we were able to identify potential genetic homologues for enzymes involved in both aromatic (dhurrin, R-prunasin) and aliphatic (linamarin) cyanogenic glycoside synthesis. Our goal of the bioinformatic analysis was to provide genetic support for whether there is a strong possibility that both cyanogenic pathway types (*i.e*., aromatic and aliphatic) are present within the American elderberry genome. This additional evidence supports our unusual (but not unprecedented) finding for the uniqueness of the American elderberry CNG profile (Appenteng et al., 2021). Taken together, these results provide additional evidence that American elderberry likely contains an unusual combination of both aromatic and aliphatic cyanogenic glycosides that are not typically found together in other plant species.

This unusual combination of cyanogenic glycosides allowed for a unique opportunity to study whether chemically distinct cyanogenic glycoside classes may correlate with a reduction of herbivory pressure to various pests within the field. Our results suggest that both the total CNG concentrations and the concentrations of each individual cyanogenic glycoside are associated with protection against a variety of biotic stressors as stronger correlations between mite pressure are seen for dhurrin and linamarin relative to amygdalin, sambunigrin (S-prunasin), and prunasin (R-prunasin) in the 2025 growing season (**Table 3C**). Additionally, the 2023 and 2024 growing season data suggests that dhurrin may be more highly associated with reduced herbivory pressure to Japanese beetles and leaf spot damage relative to the other CNGs that were present (**Tables 3A, B**). This differential association has not been studied previously, as it has not been possible to do so in plants that are known to produce only one or two specialized CNGs (Uhl and Mitchell, 2024; Piršelová and Jakubčinová, 2025). The mechanistic basis for this association may come from differences in sequestration or catabolic metabolism of each individual CNG by the pest, as some insect larvae have been reported to produce β-glucosidases that discriminate between beneficial glucosides and CNGs, but more studies are needed to address this finding and discover whether this association is directly caused by the CNGs themselves or other factors (Pentzold et al., 2014, 2015).

Correlation analyses serve as a powerful tool for scientific exploration, but it is important to note that our study has some important limitations to consider. (1) The correlations that we found within our study do not prove that cyanogenic glycosides are directly causing a reduction in herbivory damage. Our findings suggest that CNGs are present in higher concentrations in genotypes that experience less pest pressure to mites and/or Japanese beetle, but this effect could be a result of other biological factors that occur in conjunction with elevated CNGs, such as differences in stress tolerance pathways and/or genetics that are generally associated with increased accumulation of secondary metabolites. Studies in other plants indicate that cyanogenic glycosides can have a direct influence on herbivory, but more work needs to be done in American elderberry before concluding that there is a direct protective or resistant effect from CNG content against herbivory (Tattersall et al., 2001; Ballhorn et al., 2009). (2) Our study utilized fresh frozen samples from one location during one year of the field trials to determine differences in cyanogenic glycoside levels between genotypes for use in correlation analyses. It would have been ideal to directly correlate the CNG levels in each genotype with damage measurements on a per year basis from each site, but we were limited by the fresh tissue collections and funds that were available for analysis. (3) The different damage scales and observer utilized for damage measurements at HARF versus SWC may have introduced a slight unavoidable bias to the correlation analyses. However, this effect was likely minimal, as measurement recording was coordinated between the different sites and the scales were extremely similar. (4) Any significant correlations between cyanogenic glycosides levels for leaf spot damage are generalized due to combined leaf spot disease measurements. Thus, although there do appear to be weak correlations between certain CNGs and leaf spot disease, it is not clear whether higher cyanogenic potential is associated with damage occurrence induced by fungal leaf spot diseases or bacterial leaf spot diseases. Additional studies are necessary to make distinctions between fungal and bacterial disease incidence.

Stability of CNG levels in response to the environment is variable across species. Some plants (i.e, *Sorghum bicolor*) have been reported to have significant changes in CNGs diurnally and in response to abiotic stress while others (i.e, *Eucalyptus cladocalyx*) have been reported to produce CNGs constitutively across time and wounding stress (Gleadow and Woodrow, 2000; Sun et al., 2018; Emendack et al., 2018; Schmidt et al., 2018). It is well established that most cyanogenic plants have the highest cyanogenic potential in young leaves, making selection of leaves of a similar size and developmental stage necessary for accurate genotypic comparisons (Dreyer et al., 1981; Busk and Møller, 2002). Changes in American elderberry cyanogenic potential over time and in response to stress is not well described, but it has been suggested that the majority of populations that test negative, weakly positive, or strongly positive for cyanogenic potential at one time will do so again upon repeated sampling (Buhrmester et al., 2000). Thus, it is possible that environmental differences between sites may have had an unaccountable effect on our correlation analyses performed outside of the 2023 SWC site. However, with mite damage being essentially nonexistent at SWC during 2023, we concluded that extrapolation of our cyanogenic glycoside data to more robust field damage data that had been acquired during subsequent years for the same genotypes would nonetheless provide important depth to our study, as cyanogenic glycoside production is driven by genetics in combination with the environment and the genetics of our genotypes were identical at each site as a result of clonal propagation (Vetter, 2000). Cyanogenic glycoside production has been reported to be largely under genetic control in other cyanogenic species such as lima bean (Ballhorn et al., 2009).

Our study reveals that higher total concentrations of CNGs potentially have a significant correlation with reduced herbivory pressure incurred from mites and Japanese beetle in American elderberry, as significant negative correlations were seen for either the 2023 and 2025 growing seasons at SWC or HARF, respectively (**Tables 3A, C**). This is particularly interesting in relation to eriophyid mite pressure, as CNGs in other plant species are thought to become toxic when sequestration between the vacuole and intracellular components is disrupted due to mechanical injury, but eriophyid mites are classified as piercing/sucking insects that feed on host plants via the penetration of a stylet rather than mechanical chewing (Petanović and Kielkiewicz, 2010). It is possible that the mites can induce vacuolar CNG release as a result of damage or disruption to the integrity of the vacuole during feeding, as eriophyid mites directly penetrate into host epidermal cells and cause extensive leaf curling and deformation (Lindquist et al., 1996). Interestingly, a similar phenomenon was recently reported for cyanogenic glycoside activation in response to feeding by the whitefly *Bemisia tabaci*; as whiteflies rarely puncture mesophyll cells during feeding, minimal damage to plant vacuoles is incurred. These findings suggest that there may be an underlying mechanical basis through which CNGs could have a direct impact on certain piercing/sucking pests, although the mechanism is poorly understood (Walling, 2008; Easson et al., 2021). More studies are needed to determine whether the correlations that were found between CNG content and mite pressure are a result of other factors or have a direct mechanistic basis.

Individual and total cyanogenic glycoside content within each American elderberry genotype appear to have a highly significant, weak to moderate correlation (-0.20 to -0.48 Pearson correlation value depending on the individual CNG and season) with herbivory pressure incurred within the field, making CNGs within the leaf tissue a potential marker for discovering American elderberry genotypes with herbivory resistance and increasing profitability by reducing the need for pesticides (Smith, 2021). Our results indicate that the Coop, Hamilton, and East Grove genotypes may be of specific interest as varieties with natural resistance to herbivory and/or leaf spot disease, but more studies are needed to confirm this possibility (**Tables 2A–C**, **4**). Additional studies are needed to study differentiation between fungal and leaf spot damage. Hamilton, Coop and East Grove are the genotypes that would likely be of most interest to breeders, as these were the only genotypes with concentrations of cyanogenic glycosides that are likely to have functional anti-herbivory effects against generalist predators (Ballhorn et al., 2009). Most of the genotypes in our study contained relatively low CNG concentrations, suggesting that high CNG American elderberry genotypes are relatively rare, which was also reported previously in a study of cyanogenic glycoside presence in American elderberry populations without genotype distinctions (Buhrmester et al., 2000). Although genotypes with higher levels of CNGs were associated with lower incidence of herbivory, this effect plateaued at very high concentrations as the highest CNG-accumulating genotype Coop performed similarly to the second-highest CNG genotype Hamilton (**Tables 2A–C**; **Figure 3**). The limited range of the utilized damage scales may have partially played a role in this finding, as differentiating between levels of pest damage that were low can be difficult. Additionally, there could be more biological factors involved than just the CNGs themselves.

Whether there is a unique physiological purpose of very high sambunigrin (S-prunasin) concentrations is unclear for Coop and warrants additional study. Cyanogenic glycosides have been previously implicated as having potential roles in nitrogen storage and/or transport, the modulation of oxidative stress, and increased fitness to drought stress (Gleadow and Møller, 2014; Kooyers et al., 2014; Pičmanová et al., 2015). It is possible that lower levels of the other cyanogenic glycosides that are present may have biological roles even if they are not found at high enough concentrations to be associated with herbivory pressures, including regulation of nitrogen transport, ROS scavenging, and signaling effects (Cowan et al., 2022; Piršelová and Jakubčinová, 2025). Thus, it would be interesting to investigate whether Coop is more resilient to abiotic stress than the other genotypes that contain lower concentrations of CNGs and/or sambunigrin (S-prunasin) and determine whether any of the other CNGs that are present have unique biological functions in this species.

Development of cultivars from genotypes with higher cyanogenic glycoside content specifically within the leaf tissue could be a promising marker for increasing agricultural productivity. However, there may be some hesitancy by farmers to use genotypes with higher levels of leaf CNGs for agricultural purposes as consumption of these compounds is known to cause adverse effects on human health due to acute or sub-acute cyanide toxicity (Nhassico et al., 2008; Cliff et al., 2011) and very small amounts of leaf tissue may be inadvertently harvested along with the fruit. Whether high leaf CNGs correlate with increased levels of CNGs in the edible berries and flowers of American elderberry is currently unknown. However, processing is known to dramatically reduce CNG content in berry products. Additional investigation into this correlation would be valuable, as the fruit is utilized for the vast majority of American elderberry products, with interest in the flowers and flower products also gaining traction. Recent literature has reported that the CNG content within American elderberry berries and pressed juice samples is very low, being detected at levels that were not found to pose a risk to human health (Appenteng et al., 2021). In commercial American elderberry juice, no CNGs were detected at all, and processing has been shown to remove between 44-96% of total CNG content (Senica et al., 2016). These reports suggest that cyanogenic glycosides are likely to be accumulated at much higher concentrations within the leaf tissue relative to flowers and berries for American elderberry genotypes, which aligns with studies regarding cyanogenic glycoside content in European elderberry (Senica et al., 2017). Genotypes that contain low cyanogenic potential in the berries and/or juice while retaining functional levels of cyanogenic glycosides within the leaf tissue may be ideal as cultivar selections in high herbivory pressure regions. Additional research is needed to identify genotypes that have low cyanogenic potential within their edible tissues and high cyanogenic potential within the leaves.

## Data Availability

The datasets presented in this study can be found in online repositories. The names of the repository/repositories and accession number(s) can be found in the article/**Supplementary Material**.
